# Practices of caregivers when evaluating the risk of falls in the admission of older adults to nursing homes

**DOI:** 10.1590/1980-57642020dn14-040008

**Published:** 2020-12

**Authors:** Cristina Lavareda Baixinho, Maria dos Anjos Dixe

**Affiliations:** 1 Nursing School of Lisbon, Nursing Research & Development Unit – Lisbon, Portugal.; 2 Center for Innovative Care and Health Technology (ciTechcare) – Leiria, Portugal.; 3 Health School, Polytechnic of Leiria – Leiria, Portugal.

**Keywords:** nursing, accidental falls, aged, homes for the aged, cognitive dysfunction, enfermagem, acidentes por quedas, idoso, instituição de longa permanência para idosos, disfunção cognitiva

## Abstract

Falls are a complex problem for the older population residing in nursing homes. Despite recommendations, many difficulties remain in the evaluation of and systematic information on fall risk factors.

**Objectives:**

To build and validate the Scale for Practices of Identification of and Information on Fall Risk Factors in the Admission of Older Adults; to describe the practices of professionals in identifying and providing information on fall risk factors in the admission of older adults; and to associate these practices with the training, experience, and age of the caretakers.

**Methods:**

This is a methodological study. Based on a literature review, we analyzed the contexts, consulted specialists, selected indicators, and designed the scale, which was evaluated by experts. The process included a pre-test, reformulation, application, and validation.

**Results:**

The response rate was 65.52%. The validated scale has 13 items and 2 dimensions: risk factor evaluation practices and information practices; it presents good psychometric properties (α=0.913) to evaluate the risk of falls in the admission of older adults.

**Conclusions:**

Caregivers who received training during professional activities had better practices as to the provision of information to older adults about the risk of falls. The risk associated with gait and balance changes is more significant compared to the cognitive state.

## RESUMO.

As quedas são um problema complexo para a população idosa institucionalizada. Apesar das recomendações, continuam a existir dificuldades na avaliação e informação sistemática dos fatores de risco de queda.

**Objetivos:**

construir e validar a Escala de Práticas de Identificação e Informação dos Fatores de Risco de Queda na Admissão do Idoso; descrever as práticas dos profissionais na identificação e informação dos fatores de risco de queda na admissão do idoso; e associar as práticas à formação, experiência e idade dos cuidadores.

**Método:**

Estudo metodológico. A partir da revisão da literatura, foram observados os contextos, consultados peritos, selecionados os indicadores, o desenho e a avaliação da escala por peritos, o pré-teste, a reformulação, a aplicação e a validação.

**Resultados:**

A taxa de resposta foi de 65,52%. A escala validada possui 13 itens e duas dimensões: práticas de avaliação de fatores de risco e práticas de informação; apresenta boas propriedades psicométricas (α=0,913) para avaliar o risco de queda na admissão do idoso.

**Conclusões:**

Os cuidadores que receberam treinamento durante a atividade profissional apresentam melhores práticas de informação ao idoso sobre o risco de queda. Valoriza-se o risco associado às alterações de marcha e equilíbrio, comparativamente ao estado cognitivo.

## INTRODUCTION

A fall is a serious accident in the older population, with negative impacts on their functional capacity, quality of life, and mean life expectancy.^1^ ^–^ ^3^ This problem is cross-sectional in all contexts but has special relevance for older adults residing in nursing homes, among whom it is more prevalent^2^ ^–^ ^4^ and causes more severe lesions^2^ than in older people living in the community. As a result, this accident is an important source of morbidity and mortality in older adults residing in nursing homes.^1^ ^,^ ^3^ ^,^ ^5^ ^,^ ^6^

The complexity of this public health issue in nursing homes becomes more serious by the fact that their residents are more dependent and have a higher incidence of chronic diseases, polymedication, and gait changes.^7^ ^–^ ^9^

The change in environment itself and the presence of other older adults and workers are all risk factors.^2^

After a first fall or near-fall, the older individual has a higher risk of falling again, and the rate of further falls in the next year ranges from 30 to 40%.^2^ Research indicates that, after the first episode, older adults have their activities restricted by themselves or someone else due to the fear of further falls,^10,^ ^11^ which leads to dependency. Even without secondary lesions resulting from this adverse event, older adults may become afraid of falling again,^10^ which could lead to limitations in their activities. As their mobility and physical capacities decrease, the risk of falls increases.^3^

The relationship between nursing homes and falls is not exclusively associated with the fact that falls are more common and have more serious consequences in these settings. Falls among older adults living in the community is related to a risk of admission to nursing homes in the first four years after the incident.^4^

Authors and international organizations are unanimous in stating that preventive actions against this calamity are urgent, since, with population ageing, falls will increase and become a high individual, social, and economic burden.^2^ ^,^ ^4^ ^–^ ^11^ Identifying the older adults who have a high risk of falling is the first step to help professionals elaborate preventive interventions against this incident.^1^ ^,^ ^3^ ^,^ ^5^ ^,^ ^11^ The decision about whether an older individual is at risk of falling or not should be based on scientific evidence, so that the correct measures can be implemented for the right people, at the right time.^1^ ^,^ ^3^ However, risk evaluations are complex due to the multifactorial nature of this event.^1^ ^,^ ^3^ These evaluations depend significantly on the involvement of the entire team,^4^ which is necessary for the first evaluation to be carried out in the first 24 to 48 hours after admission to a nursing home.^7^ ^,^ ^8^ ^,^ ^12^

Despite these recommendations for evaluating the risk of falls in nursing homes for older adults, said evaluation is not a reality. Previous research concluded that nursing home professionals are familiar with geriatric evaluation instruments, but consider that, from the perspective of preventive care, their use is not adequate to the reality of residents.^2^ This undervaluing of their preventive capacity can contribute to their inadequate use or even to workers not using them at all.

On the other hand, the evaluation may be carried out in a period other than the admission of the older individual to the nursing home. This admission is crucial because it leads to an increased risk of falls in the first five days after the older adult becomes a resident of long-term care homes (LTCH).^2^ Additionally, communication of these risk factors within the team presents some difficulties, including communication with the older adults, who rarely receive any information on their fall risk factors in their admission to a different environment.^4^

Considering the above, the objectives of this study were: a) to build and validate the Scale for Practices of Identification of and Information on Fall Risk Factors in the Admission of Older Adults; b) to describe the practices of professionals in identifying and providing information on fall risk factors in the admission of older adults; and c) to associate these practices with the training, experience, and age of the caregiver.

## METHOD

This is a methodological study aimed to build and validate the Scale for Practices of Identification of and Information on Fall Risk Factors in the Admission of Older Adults.

The literature review found no other instrument built and allowed identifying the “state of the art” and selecting some items to build the scale, based on studies that have evaluated fall risk factors and communication. It also showed that studies carried out in nursing homes on risk factors revolve around the scales that assess these factors and not around the teams’ practices when evaluating and communicating the risk.

In addition to the literature review used to construct the scale, we analyzed the context, consulted specialists (five nurses and three doctors with more than five years of professional experience in nursing homes), selected indicators, and designed the scale, which was evaluated by experts. The process included a pre-test, reformulation, application, and validation.^13^

Each of the 21 indicators uses a 5-point Likert scale (never, rarely, sometimes, often, and always). The study considered five respondents per item.^14^

The target population of this study was formal care-givers of six nursing homes for older adults, who authorized the study. The criteria defined for the inclusion of caregivers were: professionals who worked in direct care for older adults and freely agreed to participate in the study. These formal caregivers work 40 hours a week and are responsible for providing basic human care for older adults residing in nursing homes. The study excluded nursing home professionals who held administrative positions, as well as those who only worked in house-support services or during the day.

We underline that nursing homes welcome dependent older people with and without cognitive decline. This information is important because these professionals assume an important role in satisfying the activities of daily living of this population.

The pre-test was carried out with 23 caretakers to verify their understanding of the instrument and the adequacy of the questions and of the Likert scale. An evaluation of the content and form of the instrument was carried out, with emphasis on clarity, understandability, and mean time taken to fill in the information necessary.

The questionnaire was self-administered and filled-in in the absence of the researcher. The director of the facilities informed the teams about the study, and one of the researchers went to the nursing homes to inform the objectives, methodology, and request collaboration. To ensure anonymity, boxes were placed in the nursing homes. In one of them, the participants were to insert their informed consent form; in the other, the filled-in scales. They were opened 15 days later.

The statistical treatment of data was carried out using the software *Statistical Package for the Social Sciences* (SPSS), version 23.0. The descriptive statistic techniques adopted were: absolute and relative frequency, measures of central tendency (mode, mean, and median), and measures of dispersion and variability (standard deviation, minimum, and maximum).

For statistical treatment, non-responses were replaced by the mean value of valid cases of the variable whenever the percentage of ‘non-responses’ was lower than 10% in all questionnaire items.^14^

We tested the reliability of the test by analyzing its internal consistency, resorting to the determination of Cronbach's alpha coefficient. The factorial analysis of the main components used an orthogonal rotation of the Varimax variety and the extraction of factors with face values above one. Cattell's scree test, also known as scree plot, was used to determine the number of factors that needed to be retained; the Kaiser-Meyer-Olkin (KMO) test and Bartlett's test were used to assess the quality of the correlation between the variables and test the validity of the factorial matrix.

Before the statistical test was employed to identify the relationship between variables, the Kolmogorov-Smirnov test was applied to evaluate variable distribution. Since the sample did not present a normal distribution, non-parametric techniques tested the association between the many variables of the study.^13^ ^,^ ^14^

The Mann-Whitney test, an alternative to the Student's t-test, was used for two independent samples.^14^

This study was part of the project Managing the Risk of Falls in Devices for Elders, which was approved by the Ethics Committee of the Universidade Católica Portuguesa. This investigative study complied with the ethical principles of the Declaration of Helsinki, that is, all those involved offered their informed consent, and their privacy and confidentiality were assured.

## RESULTS

The aforementioned devices were provided with 232 instruments, which received 152 responses (65.52% of the total). The sample consists exclusively of women, with a mean age of 47.02±10.3, who have worked for 12.1±8.35 years with older adults residing in nursing homes, and have been in the facility for 11.9±8.19 years.

We underline that 68% of the population started their professional activities without training that could prepare them for their work, and 66.7% attended continuing education courses. In 50.8% of cases, the courses lasted less than 150 hours, and 38.1% of them attended training courses that lasted more than 200 hours.

### Fidelity

The scale comprised 13 items, and 8 were excluded. The internal consistency of the final scale was α=0.913, which is considered excellent.^14^ If the item was excluded, the alpha value varied from 0.903 to 0.910 (Table 1). The item-total relation ranged from 0.561 and 0.730, which indicated homogeneity among the items.

**Table 1:** Pearson's correlation of the items that make up the Scale for Practices of Identifcation of and Information on Fall Risk Factors in the Admission of Older Adults and Cronbach's alpha of items with their total, without the item. Lisbon, Portugal.

Item number and content	Pearson's correlation of the total without the item	Cronbach's α without the item
1. I check whether they use mobility aids	0.613	0.907
2. I check whether they have diffculty walking	0.656	0.906
3. I observe whether their balance changes when they walk	0.730	0.904
4. I check whether they have diffculty sitting/standing up from the chair	0.656	0.907
5. I check whether they have diffculty laying down/getting up from the bed	0.724	0.904
6. I check whether they have diffculty going upstairs/downstairs	0.630	0.907
7. I check whether they know their bearings in time and space	0.629	0.907
8. I check whether they have diffculty bathing	0.626	0.907
9. I inform them about the location of the bathroom	0.599	0.909
10. I inform them about the location of the lift	0.657	0.906
11. I inform them about the use of stairs and the ramp	0.590	0.909
12. I inform them that there is a call button	0.719	0.903
13. I inform them about the motion-activated lights	0.561	0.910
Total alpha	0.913	

### Construct validity

For this scale, the KMO test result of 0.870 indicates good adequacy of the data for factorial analysis.

The result of Bartlett's test was 1149.298 for p<0.001. It tests the hypothesis that the variables are not correlated in the population.

The validity of the scale was evaluated through an exploratory factor analysis, with the extraction of factors using the technique of main components (Kaiser) with Varimax rotation. The analysis in Table 2 shows that the 13 items were organized into two factors that explain 61.803% of the variance. We highlight that the communality values presented by the items were good (0.485–0.772).

**Table 2:** Matrix of main components after the Varimax rotation of the 13 items of the Scale for Practices of Identifcation of and Information on Fall Risk Factors in the Admission of Older Adults. Lisbon, Portugal.

Item number and content	H2	F1	F2
1. I check whether they use mobility aids	0.628	0.773	
2. I check whether they have diffculty walking	0.686	0.804	
3. I observe whether their balance changes when they walk	0.720	0.793	
4. I check whether they have diffculty sitting/standing up from the chair	0.772	0.869	
5. I check whether they have diffculty laying down/getting up from the bed	0.626	0.645	
6. I check whether they have diffculty going upstairs/downstairs	0.520	0.636	
7. I check whether they know their bearings in time and space	0.500	0.500	
8. I check whether they have diffculty bathing	0.485	0.477	
9. I inform them about the location of the bathroom	0.589		0.738
10. I inform them about the location of the lift	0.564		0.665
11. I inform them about the use of stairs and the ramp	0.534		0.687
12. I inform them that there is a call button	0.729		0.799
13. I inform them about the motion-activated lights	0.679		0.818
Total explained variance		61.803	
% of variance explained per factor		33.53	28.26
Kaiser-Meyer-Olkin measure		0.870	
Bartlett's sphericity test: 1149.298; p<0.001			

Considering the content of each factor, the following name was attributed to them: F1: Practices of risk factor assessment/identification (α=0.903); F2: Practices of informing the older adult about how to prevent falls (α=0.907).

After determining the psychometric characteristics, the scale comprised 13 items grouped into two factors. The score could vary from 13 to 65 points. The better the practices, the higher the scores.

### Practices of Identification of and Information on Fall Risk Factors in the Admission of Older Adults

Regarding the practices of identification of and information on fall risk factors in the admission of older adults, and considering the median of each index r (3.5), the most frequent risk factor taken into account by caregivers is finding whether the older adult uses mobility aids: 4.43±0.88. The least valued items were “I check whether they know their bearings in time and space”, with 4.05±1.00, and “I inform them about the motion-activated lights”: 4.22±1.10.

When the mean score of each factor is analyzed, the lowest frequencies of practices are related to information (Table 3).

**Table 3:** Characterization of the older adult sample as to the identifcation of and information provided on risk factors during their admission. Lisbon, Portugal.

Item number and content	Mean	SD
1. I check whether they use mobility aids	4.43	0.88
2. I check whether they have diffculty walking	4.37	0.92
3. I observe whether their balance changes when they walk	4.32	0.88
4. I check whether they have diffculty sitting/standing up from the chair	4.37	0.87
5. I check whether they have diffculty laying down/getting up from the bed	4.36	0.85
6. I check whether they have diffculty going upstairs/downstairs	4.19	1.00
7. I check whether they know their bearings in time and space	4.05	1.00
8. I check whether they have diffculty bathing	4.37	0.94
9. I inform them about the location of the bathroom	4.34	1.05
10. I inform them about the location of the lift	4.30	1.02
11. I inform them about the use of stairs and the ramp	4.11	1.15
12. I inform them that there is a call button	4.36	0.98
13. I inform them about the motion-activated lights	4.21	1.10
Total results	47.07	12.69
F1 – Practices of risk factor assessment/identifcation	30.09	7.37
F2 – Practices of informing the older adult about how to prevent falls	16.98	5.32

SD: standard deviation.

Mann-Whitney's U test, applied to verify the profile of caregivers who were trained before starting this professional activity and/or during the professional activity, revealed that they presented a higher frequency of adequate practices than those that had no training. Trained professionals presented correct preventive behaviors and practices more often than untrained ones. Although workers who were trained before the activity had better practices, the differences were not statistically significant (Table 4).

**Table 4:** Results of Mann-Whitney's U test regarding the frequency of caregivers’ training before and after the professional activity and practices. Lisbon, Portugal.

Practice/Training before starting this professional activity		n	M. Rank	U	Z	p-value
Scale – factor 1	yes	41	67.23	1895.500	-0.054	0.957
	no	93	67.62			
Scale – factor 2	yes	44	76.83	1789.500	-1.397	0.162
	no	95	66.84			
Scale – total	yes	40	67.60	1596.000	-0.752	0.452

Those who attended professional training courses during the professional practice presented correct practices more often. These differences, however, only have statistical significance for practices and behaviors related to providing information for the older adult on how to prevent falls, as shown in Table 5.

**Table 5:** Results of Mann-Whitney's U test regarding training frequency during professional activity and practices. Lisbon, Portugal.

Practice/Training before and during the professional activity		n	M. Rank	U	Z	p-value
Scale – factor 1	yes	91	69.36	1787.000	-0.818	0.413
	no	43	63.56			
Scale – factor 2	yes	92	75.20	1683.500	-2.187	0.029
	no	47	59.82			
Scale – total	yes	86	67.52	1460.500	-1.570	0.116

## DISCUSSION

Reliability analysis shows that the 13 items in the scale are reliable, with an excellent internal consistency (α=0.913). These results show the capacity of the scale of measuring the practices of caregivers in identifying and providing information about risk factors as they receive the older adults in the nursing homes.

The caregiver's risk evaluation and their judgment when deciding which preventive measures should be introduced are central features of programs to prevent older adults’ falls in nursing homes.^2^ ^,^ ^4^ ^,^ ^15^ ^,^ ^16^ Some authors argue that this evaluation is more complex and difficult in nursing homes than in hospitals.^16^ ^,^ ^17^ However, although risk evaluations are carried out, researchers believe that they may not be valued by the team or act as an introduction to adequate preventive measures.^4^

The justification for this difficulty involves the devaluing of geriatric assessment instruments, since, from the perspective of preventive care, their use is not adequate to the reality of residents.^8^ This undervaluing of their preventive capacity can contribute to their inadequate use or even to workers not using them at all.

Research aimed at validating a protocol to manage the risk of falls in older adults residing in nursing homes even recommends sending the information on risk factors to nursing home health professionals before they welcome the older adults, so they can, in good time, plan the care and identify the potential need for products to support self-care.^2^

The results of our study suggest that, at the moment of admission, caregivers prioritize and present better practices and behaviors in risk factors related to musculoskeletal changes involving gait, muscle strength, and balance. Difficulties in gait and balance might lead to the loss of independence and directly influence self-care. As a result, caregivers become necessary for self-care and for carrying out activities of daily living.^18^

The index of the dimension “Practices of risk factor assessment/identification” with the lowest score was “I check whether they know their bearings in time and space” (4.05±1.00). This finding may indicate that cognitive decline and how it increases the risk of falls in these people could have been underestimated. Future studies should explore this issue, which is further justified by the high prevalence of falls in the older population who lives in nursing homes and have cognitive decline.^5^ ^,^ ^11^ On the other hand, cognitive decline is accompanied by changes in gait and balance and a higher risk of dependency. Risk evaluation may have been done not because of the older individual, but because of the strain that dependency can bring to caregivers.

The analysis of the subtotal score for each factor of the scale shows that both in the risk factor assessment/identification and in the provision of information about fall prevention, some practices are often used, but not always.

A study on team practices for fall prevention in hospitalized older adults found an apparent under-valuing of the information on fall risk factors and of the provision of information for older people by these professionals.^4^ This finding is probably due to the fact that falls are accepted as a normal consequence of aging and diseases,^19^ and resulting lesions are regarded as misfortune or “bad luck”.

At the time of admission, the information offered is connected to the existence of the call button and motion-activated lights, the location of the bathroom and the lift, and the use of stairs and ramps. The information provided to the older adult is important because the physical environment of nursing homes is very different from that of their houses. Environmental risks could be minimized or eliminated with behavioral changes in the population, with a positive impact on reducing risks and the prevalence of falls.^20^ ^–^ ^25^ However, to that end, the older individual must be informed about the risks and how to move safely through the facility,^24^ when obstacles are removed from transit areas.^25^ The results of the research indicate that these interventions are a priority for people with a history of falls and/or high risk of falls.^23^

Another result that deserves the attention of managers and public policies is that caregivers who had training about the phenomenon under study followed correct practices more often than those who did not. These differences, however, are only statistically significant when it comes to providing information to the older adult at the time of admission (p=0.029).

Educational programs targeted at professionals represent a positive cost-effective method to improve fall prevention strategies.^2^ ^,^ ^4^ ^,^ ^26^ Team interventions must prescribe not only the ways of approaching and controlling biophysiological and environmental risk factors,^27^ but also include practices and behaviors, especially those related to older people who have cognitive decline, so that the professionals are attentive to these practices and behaviors and can aid the weaker individuals, keeping them safe.^11^

The limitations of this study involve the intentional choice of nursing homes and of the sample, which does not allow generalizing the results. The type of instrument, how it was administered, and the time set for its completion (15 days) may have contributed for responses to be socially desirable.

Despite its limitations, the scale has an excellent internal consistency (α=0.913) and makes it possible to evaluate the practices of caregivers when assessing risk and providing information for older adults residing in nursing homes. Since the prevalence of falls is high in the first days after admission, team practices related to assessing the risk of falls must be reinforced, and the validated scale can contribute to this process. Future studies should associate the risk with the total score of the scale and each of its dimensions, the prevalence of falls in the first days after admission, and their recurrence.

## References

[1] Walker GM, Armstrong S, Gordon AL, Gladman J, Robertson K, Ward M (2016). The Falls In Care Home study: a feasibility randomized controlled trial of the use of a risk assessment and decision support tool to prevent falls in care homes. Clin Rehabil.

[2] Baixinho CR, Dixe MA, Henriques MA (2017). Falls in long-term care institutions for elderly people: protocol validation. Rev Bras Enferm.

[3] Sharifi F, Fakhrzadeh H, Memari A, Najafi B, Nazari N, Khoee MA (2015). Predicting risk of the fall among aged adult residents of a nursing home. Arch Gerontol Geriatr.

[4] Cunha LF, Baixinho CL, Henriques MA (2019). Preventing falls in hospitalized elderly: design and validation of a team intervention. Rev Esc Enferm USP.

[5] Baixinho CL, Dixe MA, Madeira C, Alves S, Henriques MA (2019). Falls in institutionalized elderly with and without cognitive decline A study of factors. Dement Neuropsychol.

[6] Bilik O, Damar HT, Karayurt O (2017). Fall behaviors and risk factors among elderly patients with hip fractures. Acta Paul Enferm.

[7] Cameron EJ, Bowles SK, Marshall EG, Andrew MK (2018). Falls and long-term care: a report from the care by design observational cohort study. BMC Fam Pract.

[8] Lannering C, Ernsth Bravell M, Midlöv P, Östgren CJ, Mölstad S (2016). Factors related to falls, weight-loss and pressure ulcers––more insight in risk assessment among nursing home residents. J Clin Nurs.

[9] Allali G, Launay CP, Blumen HM, Callisaya ML, de Cock AM, Kressig RW (2017). Falls, cognitive impairment, and gait performance: results from the GOOD initiative. J Am Med Dir Assoc.

[11] Baixinho CL, Dixe MA (2020). Practices and behaviors of professionals after falls in institutionalized elderly with and without cognitive decline. Dement Neuropsychol.

[12] Dunn JE, Furner SE, Miles TP (1993). Do falls predict institutionalization in older persons? An analysis of data from the Longitudinal Study of Aging. J Aging Health.

[13] Waltz C, Strickland O, Lenz E (2016). Measurement in Nursing and Health Research.

[14] Pestana MH, Gajeiro JN (2014). Análise de dados para ciências sociais: a complementariedade do SPSS.

[15] Borowicz A, Zasadzka E, Gaczkowska A, Gawłowska O, Pawlaczyk M (2016). Assessing gait and balance impairment in elderly residents of nursing homes. J Phys Ther Sci.

[16] Barker AL, Nitz JC, Choy NLL, Haines T (2009). Measuring fall risk and predicting who will fall: clinimetric properties of four fall risk assessment tools for residential aged care. J Gerontol A Biol Sci Med Sci.

[17] National Institute for Health and Care Excellence (2013). Assessment and prevention of falls in older people.

[18] Everink I, van Haastregt J, Tan F, Schols J, Kempen G (2018). The effectiveness of an integrated care pathway in geriatric rehabilitation among older patients with complex health problems and their informal caregivers: a prospective cohort study. BMC Geriatr.

[19] Álvarez Barbosa F, Del Pozo-Cruz B, Del Pozo-Cruz J, Alfonso-Rosa RM, Sañudo Corrales B, Rogers ME (2016). Factors associated with the risk of falls of nursing home residents aged 80 or older. Rehabil Nurs.

[20] Chacko TV, Prabha T, Muhammad GM (2017). how fall-safe is the housing for the elderly in rural areas? A cross sectional study using fall prevention screening checklist. JIAG.

[21] Maggi P, de Almeida Mello J, Delve S, Cés S, Macq J, Gosset C (2018). Fall determinants and home modifications by occupational therapists to prevent falls. Can J Occup Ther.

[22] Luk JK, Chan TY, Chan DK (2015). Falls prevention in the elderly: translating evidence into practice. Hong Kong Med J.

[23] Avin KC, Hanke TA, Kirk-sanchez N, Mcdonough CM, Shubert TE, Hardage J, Hartley G (2015). Management of falls in community- dwelling older adults: clinical guidance statement from the Academy of Geriatric Physical Therapy of the American Physical Therapy Association. Phys Ther.

[24] Sherrington C, Michaleff ZA, Fairhall N, Paul SS, Tiedemann A, Whitney J (2017). Exercise to prevent falls in older adults: an updated systematic review and meta-analysis. J Sports Med.

[25] Luck T, Motzek T, Luppa M, Matschinger H, Fleischer S, Sesselmann Y (2013). Effectiveness of preventive home visits in reducing the risk of falls in old age: a randomized controlled trial. Clin Interv Aging.

[26] Alves VC, Freitas Weslen CJ, Ramos JS, Chagas SR, Azevedo Ci, Mata LR (2017). Actions of the fall prevention protocol: mapping with the classification of nursing interventions. Rev Lat Am Enfermagem.

[27] Reis KM, de Jesus CA (2015). Cohort study of institutionalized elderly people: fall risk factors from the nursing diagnosis. Rev Lat Am Enfermagem.

